# Short-term efficacy of recombinant human GH therapy in cured acromegaly patients with GH deficiency: a single-center experience

**DOI:** 10.1530/EC-14-0132

**Published:** 2015-02-09

**Authors:** Pinaki Dutta, Bhuvanesh Mahendran, K S Reddy, Jasmina Ahluwalia, Kim Vaiphei, R K Kochhar, Prakamya Gupta, Anand Srinivasan, Mahesh Prakash, Kanchan Kumar Mukherjee, V N Shah, Girish Parthan, Anil Bhansali

**Affiliations:** Department of Endocrinology, 4th Floor, F Block, Post Graduate Institute of Medical Education and Research, Nehru Hospital, Chandigarh, 160012, India; 1 Internal Medicine, Post Graduate Institute of Medical Education and Research, Chandigarh, 160012, India; 2 Cardiology, Post Graduate Institute of Medical Education and Research, Chandigarh, 160012, India; 3 Hematology, Post Graduate Institute of Medical Education and Research, Chandigarh, 160012, India; 4 Histopathology, Post Graduate Institute of Medical Education and Research, Chandigarh, 160012, India; 5 Gastroenterology, Post Graduate Institute of Medical Education and Research, Chandigarh, 160012, India; 6 Neurosurgery, Post Graduate Institute of Medical Education and Research, Chandigarh, 160012, India; 7 Pharmacology, Post Graduate Institute of Medical Education and Research, Chandigarh, 160012, India; 8 Radiodiagnosis, Post Graduate Institute of Medical Education and Research, Chandigarh, 160012, India

**Keywords:** acromegaly, growth hormone deficiency, GH therapy

## Abstract

The effectiveness and short-term safety of recombinant human GH (r-hGH) in acromegaly patients with GH deficiency (GHD) after treatment are not well established. The study includes ten subjects with acromegaly who had GHD treated with r-hGH for 6 months. Control groups consisted of ten age-, gender-, and BMI-matched healthy subjects and ten active acromegaly patients who were treatment naïve. Body composition, quality of life (QoL), muscle strength, lipid profile, and cardiovascular risk factors were assessed in all subjects at baseline, and the same parameters were reassessed after 6 months of therapy with r-hGH in acromegaly with GHD. Repeat magnetic resonance imaging of the sella was performed in treated subjects. Optical colonoscopy was done and biopsies were taken from multiple sites for proliferation indices (Ki67). The median duration of GHD was 17.8 months and dose of r-hGH administered was 5.7±1.5 μg/kg per day. There was improvement in bone mineral content (*P*=0.01), bone mineral density (*P*=0.04), muscle strength (*P*<0.001), total cholesterol (*P*=0.003), high-density cholesterol (*P*<0.001), and QoL – score (*P*=0.005), and reduction in low-density cholesterol (*P*=0.003) and triglyceride (*P*=0.004) after treatment. There was no change in lean body mass, total body fat, hsCRP, lipoprotein (a), and fibrinogen levels. There was a modest increase in plasminogen activator inhibitor 1 (*P*=0.002), but it was lower compared with healthy controls and treatment naïve acromegalics (*P*=0.007). Six month-r-hGH therapy improves body composition, atherogenic lipid profile, QoL, and muscle strength in GHD patients who had acromegaly. Long-term prospective studies are needed to evaluate the effect of r-hGH therapy in these patients.

## Introduction

Acromegaly is most commonly caused by a growth hormone (GH)-producing pituitary tumor and is potentially life-threatening if untreated. Early diagnosis and treatment of acromegaly result in increased longevity and better quality of life (QoL) (1). However, successful treatment of acromegaly either by surgery, radiation or combinations of different treatment modalities results in GH deficiency (GHD) in nearly 30–60% of patients (2, 3). The prevalence of GHD increases as the duration of radiotherapy increases in acromegaly patients (3). The prevalence of GHD is higher in treated acromegaly patients as compared with patients with nonfunctioning pituitary adenomas treated with similar modalities (15% vs 5%) (4). GHD in adulthood is characterized by abnormalities in body composition and physical and psychological functions. Studies have shown that GHD in non-acromegalics subjects could increase the cardiovascular risk and mortality and impair QoL, even when other pituitary hormones are intact or properly replaced (4, 5, 6). In addition, GH replacement in these subjects has been shown to reverse many of the abnormalities (7).

There is a paucity of data on recombinant human GH (r-hGH) replacement therapy in acromegaly patients with GHD after cure of the disease. There are three open-label studies and one randomized control trial, one large retrospective cohort study, and one recently published meta-analysis which attempted to address this issue (8, 9, 10, 11, 12). However, these studies are limited by incomplete evaluation of cardiovascular parameters of retrospective nature, heterogeneous data quality, and inclusion of patients who were drug naive and sub drug naive (8). This study was planned to assess the changes in the novel cardiovascular risk parameters and QoL in acromegaly patients with GHD following r-hGH replacement.

## Patients and methods

This was a 6-month open-label intervention study conducted from January 2011 to January 2013. Ninety-nine acromegaly patients who were under follow-up in the pituitary clinic of Postgraduate Institute of Medical Education and Research, a tertiary care institute in Chandigarh, India, were screened for GHD. The study was approved by the Institute's Ethics Committee and written informed consent was taken from all subjects.

The diagnosis of acromegaly was based on nonsuppressible GH (>1 ng/ml) following 2-h oral glucose tolerance test (OGTT) with 75 g of anhydrous glucose. All recruited patients underwent surgery and or radiotherapy at our institution. After treatment, patients who had suppressible GH OGTT (<1 ng/ml) were subjected to insulin-induced hypoglycemia (IIH). GHD was defined as peak GH level ≤3 ng/ml during IIH and insulin-like growth factor 1 (IGF1) level <2 s.d. below age-specific normal range (13).

Patients who were on pharmacological therapy for acromegaly, unstable cardiovascular disease, uncontrolled hypertension or diabetes mellitus, pregnancy or lactation, previous history or active malignancy, chronic kidney disease, chronic liver disease, unexplained anemia, and active disease were excluded. All subjects had normal colonoscopy while female subjects had normal mammography and papanicolaou cervical smear at baseline. Patients with other concomitant anterior pituitary hormone deficiencies were started on specific hormone replacement therapy for at least 3 months before their enrolment into the study. None of the patients were on transdermal testosterone or estrogen therapy. Ten (five males and five females) acromegaly patients with GHD were included in the study. The control group consisted of age-, gender-, and BMI-matched ten healthy volunteers (healthy control group (HCG)) and ten active acromegaly patients who were treatment naïve (acromegaly control group (ACG)). Only baseline parameters were analyzed in the control groups.

Baseline fasting blood samples were taken for estimation of hormonal parameters, including IGF1 and cardiovascular risk markers in the form of high-sensitivity C-reactive protein (hsCRP), complete lipid profile, plasma fibrinogen, lipoprotein (a) (Lp(a)), fasting plasma glucose, insulin, and HbA1c. IIH was done to define GHD as per standard protocol.

Muscle strength was assessed in the nondominant hand after due acquaintance with Jamer's dynamometer and an average of three measurements was taken as the final reading as kilogram force. Bone mineral content (BMC), bone mineral density (BMD), total body fat (TBF), and lean body mass (LBM) were calculated by dual-energy X-ray absorptiometry (DXA; Hologic, Discovery-A, Bedford, MA, USA) by a single observer (M Prakash) blinded to the patients' information. The DXA was performed for whole body for body composition and three sites (hip, distal radius, and spine) to find out BMD values and *T* and *Z* scores. The precision error of the DXA machine was ±1% if it was repeated within 24 h and 1.0–2.5% if it was repeated after 2–6 months. To determine cardiac indices, echocardiography and tissue Doppler (Philips i33, 3D ECHO) were performed by another observer (K S Reddy) blinded to the patients' information. Full-length optical colonoscopy was carried out to evaluate the presence of colonic polyp and biopsies from multiple sites including polyps were obtained (R K Kochhar) for proliferation indices (Ki67, K Vaiphei). Magnetic resonance imaging (MRI) of sella was performed at baseline and at the end of the study to identify any changes in residual tumor or appearance of new tumor. All relevant tests were repeated at the end of the study.

QoL was assessed by questions on life satisfaction–hypopituitarism questionnaire (QLS-H) (14). This scoring system was translated in our local language and validated before application. The total QLS-H score was subsequently obtained by adding the individual item scores of the nine dimensions and could range from −180 (representing very low satisfaction) to +180 (representing very high satisfaction) (Supplementary File 2, see section on supplementary data given at the end of this article).

### Laboratory methods

Serum and plasma samples were collected and stored at −80 °C. IGF1 was estimated by enzyme immunoassay using a commercially available kit (IA-2947; DRG, Marburg, Germany). Minimal detectable IGF1 concentration by this assay was 0.15 ng/ml. Age-specific normal ranges for IGF1 values are provided in Supplementary Table 1, see section on supplementary data given at the end of this article. GH and plasma insulin were estimated by electro chemiluminescence immunometric assay (Cobas 800, Roche–Hitachi) with minimal detection limit of ≤0.05 ng/ml and 0.2 μU/ml respectively. The inter- and intra-assay coefficients of variation (CV) were <5%. hsCRP was measured by enzyme immune-assay (Biocheck, Inc., Foster City, CA, USA) with an inter-assay CV <5% and lowest detection limit of <0.1 mg/l. Lp(a) was measured by latex agglutination enhanced immunoturbidometric assay (Agappe Diagnositic, Kerala, India) with the upper normal limit being upto 30 mg/dl in healthy individual. There was no assay interference. Plasma fibrinogen was measured by electromagnetic mechanical system, STA–Fibrinogen (DiagnosticaStago, Paris, France) with normal plasma fibrinogen level being 1.5 g/l. Plasminogen activator inhibitor 1 (PAI1) was assessed by enzyme-linked immune-assay by commercially available kit (108891; Abcam, Cambridge, UK) with minimum detectable limit 300 pg/ml and intra and inter-assay CV of 4.7 and 7.2% respectively. Plasma glucose level was estimated by glucose oxidase method and HbA1c by HPLC (Bio-Rad, D10).

### r-hGH replacement protocol

After initial evaluation, the subjects were given r-hGH subcutaneously daily at bed time considering age, gender, and use of gonadal steroids. For males ≥50 years of age, the dose was 3 μg/kg per day, for males <50 years of age the dose was 4 μg/kg per day. For females <50 years and not on estrogen replacement, the dose was 5 μg/kg per day and if <50 years of age with estrogen replacement the dose was 6 μg/kg per day. The subjects were followed up at 1, 3, and 6 month intervals after initiating GH replacement therapy. The dosage of GH was adjusted according to the IGF1 levels during follow-up visits at 6 weeks and 3 months. The target level of IGF1 was set at the middle of the age-adjusted range.

The efficacy of r-hGH after 6 months of treatment was measured in the form of changes in echocardiographic and tissue Doppler parameters, change in muscle strength, biochemical markers, and QoL as compared to baseline parameters. Safety outcome were assessed by any adverse events directly related to GH therapy.

### Statistical analyses

The primary outcome was to measure the change in Lp(a) before and after treatment with r-hGH. The study was designed to detect difference of four in the Lp(a). A power of 80% and *α* error of 0.05 were considered as significant. The sample size was calculated as 10 to fulfil all the parameters. Descriptive measures such as mean, median, mode, CIs, and percentiles were analyzed using SPSS version 21. Correlation, regression, *t*-test (paired), and *χ* ^2^-test were also applied wherever appropriate. Continuous variables were analyzed with correlation, regression, and *t*-test (paired). Categorical variables were analyzed by *χ* ^2^-test. Nonparametric variables between the control and test group were analyzed using Mann–Whitney *U* test. Parametric variables between the control and test group were analyzed using *t*-test for equality of means.

## Results

Comparative estimates of various parameters in the control (HCG) and test groups at baseline and those after GH treatment in the test group are given in Tables 1, 2 and 3. The mean dose of r-hGH administered was 5.7±1.5 μg/kg per day. The dose requirement to achieve comparable IGF1 level was marginally higher in females as opposed to male patients.

### Body composition

The mean BMC in the HCG and test group were 2126.8±355.5 and 2480.6±488.6 g respectively, and it increased to 2562.2±455.7 g (*P*<0.01) after 6 months of r-hGH therapy in the test group. Mean BMD improved from 1.09±0.09 to 1.109±0.09 g/cm^2^ in the test group after treatment (*P*<0.04). Similarly, TBF level also showed improvement. There was no significant change in LBM following treatment.

### Muscle strength

**Figure 1:**
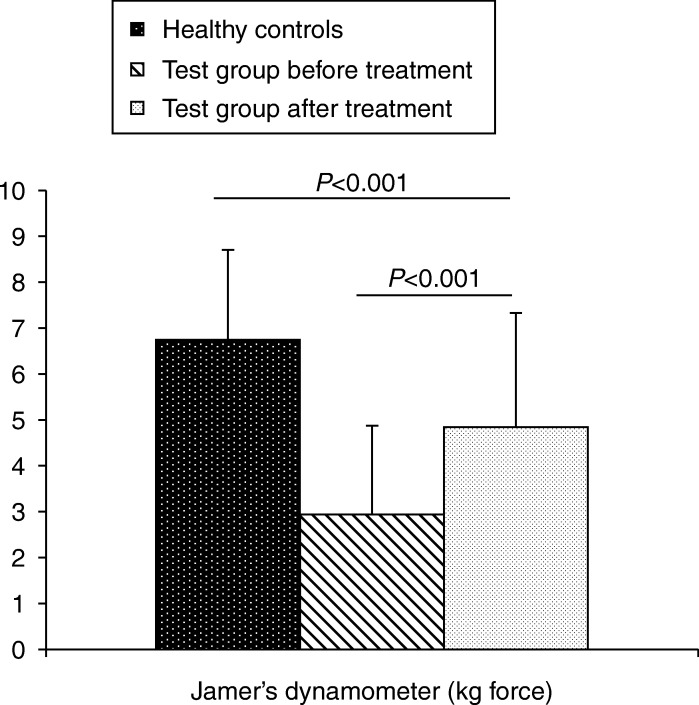
Bar diagram comparing the mean muscle strength between the groups.

Mean±s.d. muscle strength in the HCG was 6.7±2.0 while it was 2.9±1.96 in the test group before treatment, which improved to 4.8±2.52 kg force after treatment (*P*<0.001, Fig. 1).

### Biochemical parameters

**Figure 2:**
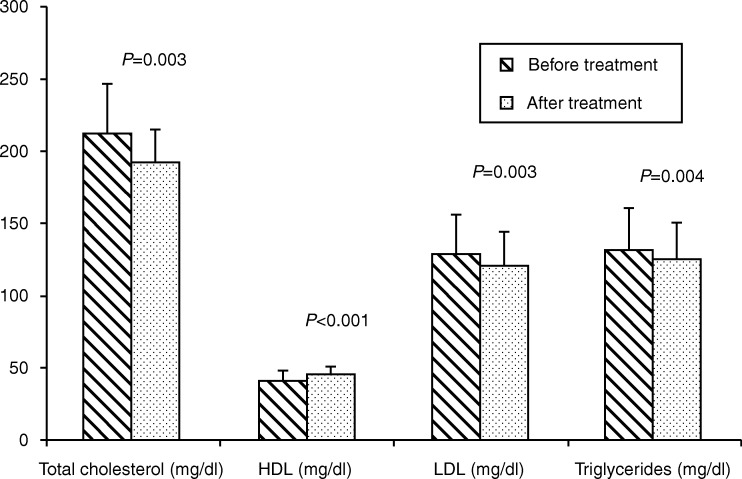
Bar diagram comparing the lipid profile in the test group before and after therapy.

The IGF1 SDS before treatment was −2.5 (0.4) which improved to 1.7 (1.5, *P*≤0.005), which was even better than healthy controls (−0.7 (0.6), *P*=0.001). At baseline, subjects in the test group had significantly higher LDL as compared with healthy controls. The level of total cholesterol was higher and HDL was lower. However, after 6-month r-hGH therapy, total cholesterol and LDL level remained higher in the control group. Mean HDL in the test group increased significantly following treatment (41.7±6.1 to 45.7±5.2, *P*<0.001, Fig. 2). In addition, Lp(a) also decreased in the test group, although the baseline levels were similar to those in healthy controls.

### High-sensitivity C-reactive protein

Mean hsCRP levels were comparable between the healthy controls and the test group, and following intervention, there was a marginal decrease from 0.05 (interquartile range (IQR) 0.01–0.18) to 0.02 (IQR 0.01–0.16, *P*=0.60) in the test group.

### Coagulation parameters

Mean fibrinogen levels were comparable between the healthy control and test group at baseline. There was a decrease in the mean fibrinogen in the test group post treatment (*P*=0.005). At baseline PAI1 levels were significantly lower in the test group as compared with healthy controls (8.4±4.4 ng/ml vs 23.8±5.9 ng/ml, *P*<0.001). Following treatment with r-hGH, the PAI1 level increased from 8.4±4.4 to 12.65±5.10 ng/ml (*P*<0.002) in the test group. However, it was much lower than the levels observed in patients with treatment-naïve active acromegalics and healthy controls (*P*=0.007, Table 3).

### Quality of life

**Figure 3:**
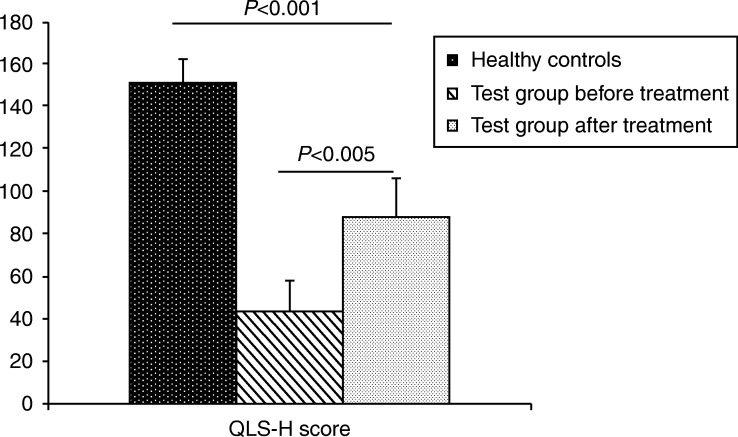
Bar diagram showing the median QLS-H score between the groups.

The median (IQR) QOL score in the control group was 153 (143–149). The median (IQR) QoL in the test group before treatment was 45 (36–55.25) which improved to 84.5 (79–100) post treatment (*P*=0.005, Fig. 3) (Supplementary File 2).

### Echocardiographic parameters

Mean ejection fraction (EF) in the control and test groups were 63% (58–65.5) and 51.5% (49.25–52.75) respectively, which improved to 58% (56–65) in the test group after treatment (*P*<0.005). The mean left ventricular (LV) mass was comparable between the cases and controls at baseline; however after treatment it increased to 136.6±20.61 g in the test group (*P*<0.006), reaching a mass equivalent to that of treatment-naïve active acromegaly patients.

The end-diastolic ‘E’ wave was worse in the test group and reached a comparable value as that of the control group following treatment (from 0.73, range 0.62–0.79 to 0.8 range 0.8–0.89, *P*<0.03). Diastolic dysfunction, depicted in the form of end-diastolic time, improved from baseline following treatment, from 204 (range 193–219.5) to 186 (range 166–178.95 ms, *P*=0.005). Other echocardiographic parameters did not improve much in the intervention group.

As expected, the treatment-naïve active acromegaly patients had significantly higher IGF1, marginally elevated but insignificant LDL, triglyceride (TG), hsCRP, BMC, BMD, TBF, LBM, and LV-mass as compared with acromegaly patients with GHD at baseline and even after treatment. The fibrinogen level, Lp(a), and EF were similar and insignificantly lower and muscle strength was higher (*P*=0.03) as compared with baseline and post-treatment value in test group (Table 3).

### Predictors of response

The change in IGF1 level predicted the change in body composition (*r*=0.60, *P*=0.006), improvement in LDL (*r*=0.4, *P*=0.04), and QoL (*r*=0.68, *P*=0.03). The lower the peak GH response to IIH, the better was the improvement in muscle strength and QLS-H. There was no difference in the response(s) of patients who were treated for partial or pan hypopituitarism.

### Side effects and safety parameters

Side effects were minimal and were not of much clinical significance. Three patients developed side effects in the form of arthralgia, edema, and carpel tunnel syndrome respectively, which were self-limiting. Proliferation indices on colonic mucosal biopsy remained unchanged (<5%) and there was no recurrence/re-growth of pituitary tumor on MRI.

## Discussion

The study shows an improvement in body composition, atherogenic lipid profile, muscle strength, echocardiographic parameters, and QoL without any change in coagulation parameters following 6 months of r-hGH therapy in acromegaly patients with GHD. The short-term GH replacement is not associated with tumor re-growth and colonic epithelial changes.

GHD in adults is characterized by abnormalities in body composition, physical and psychological function, and increased mortality even if all other pituitary hormones are intact or adequately replaced (6, 17, 18). r-hGH replacement therapy in adult with GHD (AGHD) due to various causes other than post-treatment acromegaly is well established (14). GH replacement normalizes most of the signs and symptoms of GHD, and may reduce the excess vascular risk in patients with AGHD (4, 7, 19). Although the visceral obesity in AGHD is one of the factors related to increase all cause and cardiovascular mortality, it is not conclusive. Studies examining these issues in patients with acromegaly who are cured and have GHD are either scant or fraught with incomplete evaluation (8, 9, 10, 11, 12, 13). A recently published large retrospective pharmaco epidemiology study by Tritos *et al*. **has shown long-term safety and efficacy of r-hGH in acromegaly patients with GHD. However, the study has following limitations: retrospective nature, heterogeneous data quality, inclusion of patients who were drug naive and sub drug naive and patients who were exposed to r-hGH for 75% of observation period, and no evaluation of novel cardiovascular risk parameters. Similarly, in this study patients with low IGF1 without GH dynamic on provocation tests were also included, despite the fact that in 30–40% there is dissociation between IGF1 and GH values post acromegaly. This study also failed to address the effect of previous GH excess on cardiovascular outcome (8).

One of the salient features of AGHD is the increase in peri-visceral fat by 7–10%. Peri-visceral fat overexpress the enzyme 11β-hydroxy steroid dehydrogenase 1, which converts cortisone to cortisol, and cortisol is a direct regulator of PPARγ (15, 16, 17, 18). This perpetuates adipogenesis and the release of various inflammatory cytokines. This could be one of the plausible mechanisms of increase in cardiovascular morbidity and mortality in patients with GHD (20). The r-hGH replacement has been demonstrated to reduce peri-visceral fat, promote LBM and, in turn, increase muscle strength in the patients with AGHD (21). The data on the effect of r-hGH therapy on body composition in patients with cured and GH-deficient acromegaly are conflicting (4, 7, 8, 9, 10, 12, 15, 16). In this study, there was a significant decrease in weight, BMI, and TBF after 6 month-r-hGH replacement. However, there was no significant improvement in LBM in our study, which is in contrast to the previous studies (6, 9, 13, 22, 23, 24).

In this study, there was significant increment in the BMD and BMC after 6-month r-hGH replacement therapy. Previous studies suggest that a minimum of 12 months is necessary to detect any difference in BMD. Most of the studies evaluating the effects of GH on BMD show a decrease at 6 months, indicating excessive resorption (12). We observed a significant improvement probably because of more severe involvement of bones and the effect has not been studied in acromegaly patients with GHD previously. Despite higher GH and IGF1 levels in patients with acromegaly, they are at increased risk for low BMD. Paradoxically, fracture risk is increased in acromegaly patients even with increased BMD or BMC. So the improvement in bone parameter may not reflect the decreased fracture risk. On the contrary, patients with GHD due to nonsomatotroph tumors have preserved BMD and BMC, and r-hGH replacement therapy improves the BMD by the end of 1 year (22, 25). It would have been prudent to assess bone turn over markers before during and after GH replacement therapy in acromegaly patients with GHD.

AGHD is associated with alterations in lipid profile characterized by an increase in total cholesterol, LDL-C, and TG and low levels of HDL-C. This is possibly due to a decrease in lipoprotein lipase activity and increase in hormone-sensitive lipase activity (26, 27). This study observed a decrease in TGs, cholesterol, and LDL-C levels and an increment in HDL-C after 6-month r-hGH replacement therapy, similar to the recent study by Tritos *et al*. (8). Findings suggest that r-hGH replacement has a beneficial effect on the atherogenic lipid profile and thus might significantly reduce the cardio vascular disease risk. A randomized, placebo control study documented the reversal of atherogenic lipid profile, which go hand-in-hand with improvement in body composition following r-hGH in acromegaly patients who were GHD (9).

The data on the effect of GH replacement in patients of acromegaly who developed GHD on novel cardiovascular risk factors are scanty, only hsCRP and fibrinogen have been well studied. In addition to conventional risk factors for cardiovascular disease, we also studied the effect of r-hGH on Lp(a) and PAI1. No study has addressed the effect of r-hGH on these parameters in acromegaly patients with GHD. Lp(a) is an independent atherogenic lipoprotein that can be thrombogenic and may be used as a plasma marker for individuals at risk for cardiovascular events (28). AGHD has been shown to be associated with high Lp(a) that might add further risk to the already existed high cardiovascular risk (29). Studies have shown that treatment of childhood GHD leads to an increase in Lp(a) (28, 29). However, Lp(a) rise with GH therapy in AGHD is not known. We observed a significant reduction in the Lp(a) following treatment with r-hGH. Whether this decrease in Lp(a) reduces long-term cardiovascular risk is not known. Furthermore, Lp(a) concentration is highly inheritable and its physiological function(s) has not been clearly established. Data suggests that it acts in concert with the plasminogen system, as its gene is derived from a duplication of the plasminogen gene (30).

Patients with active acromegaly as well as AGHD are documented to have elevated levels of fibrinogen and PAI1, which are associated with advanced atherosclerosis, new cardiovascular events, and increased risk of atherothrombosis (31). Also, this is considered to pre-date recurrent myocardial infarction. Long-term treatment with GH in AGHD patients has been shown to reduce PAI1 activity (31). In contrast, in patient with active acromegaly, PAI1 activity is increased (32). In this study, we observed a modest increase in the PAI1 after r-hGH replacement, although it was still lower than the healthy controls, suggesting that the increment may not adversely affect the coagulation profile. In line with previous studies, there was significant decrease in fibrinogen levels in the test group after GH replacement. Overall, GH replacement has a beneficial effect on the blood coagulation parameters. This could be a direct effect of GH itself, or it may be secondary to the favorable changes in body composition.

Previously published placebo-controlled studies in AGHD in non-acromegalics and one randomised, placebo-control study in acromegaly subjects with GHD reported that GH replacement reduces hs-CRP (9, 32). We observed no difference in hs-CRP levels between the test and control groups at baseline and after treatment. It could be due to the short duration of our study, and different assay method used.

QLS-H module to assess QoL is well-documented and is considered as a sensitive tool for assessment in hypopituitarism (33, 34, 35, 36, 37). Post-treatment acromegaly patients with GHD had diminished QoL compared with patients with acromegaly whose peak GH was within normal range (38). The improvements in QoL with GH replacement in acromegaly patients with GHD post treatment are variable. Results from two open-label trials had shown no improvement, while Miller *et al*. and we observed improved QoL with GH replacement therapy. The improvement could be attributed to generation of neurotransmitters, restoration of normal tissue hydration, and somatic changes in body composition, cardiovascular and reproductive health, skin turgor, and improvement in exercise capacity (37).

Muscle strength was assessed by Jamer's dynamometer, which is a well-validated tool (35, 36). In our study, we found a significant improvement in muscle strength following treatment with rGH. This can be attributed to a small, but insignificant increase in LBM that was observed in the study population. This requires region-wise measurement of muscle mass by sensitive tools.

Acromegaly and GHD lead to specific structural and functional cardiac alterations. In active acromegaly, a specific myocardial change develops, which is characterized by concentric left ventricular hypertrophy (LVH) and LV systolic and diastolic dysfunction (39). We observed that the patients in the GHD group at baseline had significantly lower EF compared with normal controls. The EF improved to near normal with GH replacement. Similar observation was made with LV mass in the treated group in a previous study (40). Although there can be some changes in cardiac parameters that occur in many patients, they do not necessarily result in cardiomyopathy. Whether this increase is beneficial or detrimental needs long-term follow-up. Increased LV mass with GH treatment could be a problem in such patients because many of the acromegalic patients die of cardiovascular complications. The increase in LV mass leads to phenotypic changes in membranous proteins conduction system and structural uncoupling of myocytes, resulting in an increased number of re-entrant events (41). Even short-term GH–IGF excess leading to increased LV mass is detrimental by causing diastolic dysfunction, which in turn has been directly related to increased mortality even with normal systolic function (41, 42, 43). However, in our study diastolic dysfunction improved following GH replacement, eliminating differences with controls.

None of the patient developed diabetes mellitus or impaired glucose tolerance. The homeostasis model assessment-insulin resistance (HOMA-IR) and HbA1c did not changed significantly following treatment (data not shown).

No cardiovascular morbidity and mortality were found in our study unlike those reported in a relatively short open-label study by Norrman *et al*. (10) and a large long-term study by Tritos *et al*. (8), in which the all-cause mortality and cardiovascular mortality were significantly increased. The difference in our result could be because of short duration of study, and none of our patients had co-morbidities such as hypertension, diabetes mellitus, and established coronary artery disease (8, 10). A word of caution should be made that GH therapy might be inappropriate in patients with elevated cardiovascular risk. Also, we did not find any recurrence or re-growth of pituitary adenomas, colonic polyps, increased proliferation indices, or atypia within 6-month therapy. However, this is too short to evaluate the tumor growth or malignancy development even in acromegaly.

Osteoarthritis changes in acromegalic patients with long duration of disease is a well-known phenomenon due to exposure to high GH and IGF1 levels. This complication may progress even in cured patients during the course of the disease without any symptoms. In our study, three out of ten treated patients developed side effects in the form of arthralgia, edema, and carpel tunnel syndrome. With longer duration of GH therapy these may further worsen and more number of patients may develop this.

Some of parameters in the test group did not improve following r-hGH therapy, this could be due to short duration of r-hGH therapy. The other explanation could be infinitely varied genotypes (pharmacogenomics) and imperfect replacement of other pituitary hormone deficiency in real-life practice. The strength of our study include prospective nature and evaluation of changes in cardiovascular risk factors, especially Lp(a), PAI1, and echocardiography during r-hGHD therapy. The limitations of our study are small sample size, open-label nature, nonrandomization of patients, and short duration of r-hGH therapy which does not allow to draw any firm conclusion about the side effects. A better comparator group could have been non-acromegaly patients with AGHD supplemented with r-hGH and acromegaly patients with AGHD who are untreated to segregate-specific effects of GH from natural course of cured acromegaly patients.

In a study by Van Bunderen *et al*. (4), GHD acromegaly patients did worse than GH sufficient-treated acromegaly patients and acromegaly patients with active disease. However, the open-label nature of our study, small sample size, short duration of treatment period, and lack of large randomized control trials make firm conclusions impossible. Thus, suggesting GH replacement as a standard of care in GHD acromegaly patients in future may not be appropriate. It can be logical to suggest GH therapy only in younger patients with relatively short duration of disease again with caution.

In summary, 6-month GH replacement therapy improved body composition, atherogenic lipid profile, QoL, muscle strength, and cardiac function in post-treatment acromegaly with GHD.

## Supplementary data

This is linked to the online version of the paper at http://dx.doi.org/10.1530/EC-14-0132.

## Author contribution statement

P Dutta, B Mahendran, K S Reddy, J Ahluwalia, K Vaiphei, R K Kochhar, P Gupta, A Srinivasan, M Kumar, K K Mukherjee, V N Shah, G Parthan and A Bhansali have contributed equally to this article.

## Figures and Tables

**Table 1:** Comparison of baseline, clinical, and laboratory parameters in healthy control group (HCG) and subjects receiving hGH replacement therapy.

	**Healthy control** (*n*=10)	**Test group** (*n*=10)	***P* value**
Male/female	5:5	5:5	NS
Mean age (years)	43.4 (9.5)	44.9 (9.2)	NS
BMI (kg/m^2^)	26.7 (3.6)	26.9 (3.5)	0.89
Mean weight	70.0 (62.8–75.0)	70.0 (64.2–76.5)	0.85
Mean duration of symptoms (years)	–	4.7	NA
Mean duration of cure (months)	–	17.8	NA
Hypothyroid	0	8	
Hypogonadal		5	
Hypocortisolic	0	5	
Estrogen replacement		1	
Median peak GH on IIH (IQR) (ng/ml)	10.2 (10–18.2)	0.6 (0.09–1.2)	
IGF1 (ng/ml)	129.3 (105.7–146)	32.4 (26.6–57.2)	<0.001*
IGF1 SDS^a^	−0.7 (−0.6)	−2.5 (0.4)	0.001*
Total cholesterol (mg/dl)	186 (19.6)	212.6 (34)	NS
LDL (mg/dl)	101 (16.5)	129.8 (26.2)	0.03
HDL (mg/dl)	42 (8.2)	41.71 (6.1)	NS
Triglyceride (mg/dl)	140 (6.4)	131.8 (28.2)	NS
Lipoprotein (a) (mg/dl)	20.9 (17.3–23.9)	21.9 (17.1–39.1)	0.73
hsCRP (mg/l)	0.08 (0.03–0.18)	0.05 (0.01–0.18)	0.43
Fibrinogen (g/l)	2.9 (2.8–3.3)	3.1 (2.9–3.1)	0.43
PAI1 (ng/ml)	23.8 (5.9)	8.4 (4.5)	<0.001*
Muscle strength (kg force)	6.7 (2.0)	2.9 (1.9)	<0.001*
BMC (g)	2126.8 (355.5)	2480.6 (488.6)	0.08
BMD (g/cm^2^)	1.09 (0.06)	1.09 (0.09)	0.84
TBF (g)	28 347.7 (10 189.6)	32 356.4 (10 432.5)	0.39
LBM (g)	40 962.1 (7322.6)	37 072 (6545.7)	0.22
QLS-H score	153 (143–149)	45 (36.0–55.25)	<0.001*
Ejection fraction (%)	63 (58–65.5)	51.5 (49.2–52.7)	0.01
LV mass (g)	131.8 (29.05)	129.4 (22.1)	0.83
EDT	171 (155.2–189.7)	204 (193–219.5)	0.005
E:A ratio	1 (1–1.1)	1.02 (0.8–1.2)	0.79
E:E′ ratio	0.09 (0.09–0.10)	0.08 (0.07–0.08)	0.01

*P value <0.05 – statistically significant median (IQR). GH, growth hormone; IIH, insulin-induced hypoglycemia; IGF1, insulin-like growth factor 1; IQR, interquartile range; hsCRP, high-sensitivity C-reactive protein; PAI1, plasminogen activator inhibitor; BMC, bone mineral content; BMD, bone mineral density; LBM, lean body mass; QLS-H, questions on life satisfaction–hypopituitarism; LV mass, left ventricular mass; EDT, end-diastolic time; E:A, ratio is the ratio of the early (E) to late (A) ventricular filling velocities; E:E′, ratio of transmitral Doppler early filling velocity to tissue Doppler early-diastolic mitral annular velocity; TBF, total body fat.

a IGF1 SDS before treatment (median −2.7, 25th percentile=−2.9 and 75th percentile=−2.2). In healthy control (median −8.0, 25th percentile=−1.2 and 75th percentile=0.50).

**Table 2:** Comparison of various parameters in the test group at baseline and following GH therapy.

	**Baseline** (*n*=10)	**Following intervention** (*n*=10)	***P* value**
IGF1 (ng/ml)	38.7±18.4	240.8±57.5^a^	0.005
IGF1 SDS score^b^	−2.5 (0.4)	1.7 (1.5)	<0.005
Total cholesterol (mg/dl)	212.6 (34)	193.0 (22.0)	0.003
LDL (mg/dl)	129.8 (26.2)	121.1 (24.0)	0.003
HDL (mg/dl)	41.7 (6.1)	45.7 (5.2)	<0.001
Triglyceride (mg/dl)	131.8 (28.2)	125.8 (24.9)	0.004
Lipoprotein (a) (mg/dl)	21.9 (17.1–39.1)	17.6 (15.2–34.6)	0.005
hsCRP (mg/l)	0.05 (0.01–0.18)	0.02 (0.01–0.16)	0.600
Fibrinogen (g/l)	3.1 (2.9–3.1)	2.85 (2.8–2.9)	0.005
PAI1 (ng/ml)	8.4 (4.5)	12.7 (5.1)	0.002
Muscle strength (kg force)	2.9 (1.9)	4.8 (2.5)	<0.001
BMC (g)	2480.6 (488.6)	2562.2 (455.7)	0.01
BMD (g/cm^2^)	1.09 (0.09)	1.1 (0.09)	0.04
TBF (g)	32 356.4 (10 432.5)	30 463.9 (9018.8)	0.004
LBM (g)	37 072 (6545.7)	37 228 (6199.7)	0.70
QLS-H score	45 (36.0–55.25)	84.5 (79–100)	0.005
Ejection fraction (%)	51.5 (49.2–52.7)	58 (56–65)	0.005
LV mass (g)	129.4 (22.1)	136.6 (20.6)	0.006
EDT (ms)	204 (193–219.5)	186 (166–188)	0.005
E:A ratio	1.02 (0.8–1.2)	1.05 (1.0–1.2)	0.12
E:E′ ratio	0.08 (0.07–0.08)	0.07 (0.07–0.08)	0.54

a Presented as mean±s.d., rest of the data presented as median (IQR).

b IGF1 SDS before therapy (median −2.7, 25th percentile=−2.9 and 75th percentile=−2.2). IGF1 SDS after therapy (median 1.1, 25th percentile=0.6 and 75th percentile=2.1).

**Table 3:** Comparison of various parameters between GHD and treatment naïve active acromegaly control group (ACG) at baseline.

	**GHD acromegaly baseline** (*n*=10)	**Treatment-naïve active acromegaly patients** (ACG)	***P* value**
Male/female	5:5	5:5	NS
Mean age (years)	44.9 (9.2)	35.3 (4.0)	NS
BMI (kg/m^2^)	26.9 (3.5)	27.8 (2.5)	0.81
Mean duration of symptoms (years)	4.7	4.3	NS
Mean duration of cure (months)	17.8	–	NA
Hypothyroid	8	7	NS
Hypogonadal	5	5	NS
Hypocortisolic	5	3	NS
Estrogen replacement	1	2	NS
Peak GH on IIH (ng/ml)	0.6 (0.09–1.2)	–	–
IGF1 (ng/ml)	38.7±18.4	760±50.2^a^	<0.001
Total cholesterol (mg/dl)	212.6 (34)	225 (10.2)	0.69
LDL (mg/dl)	129.8 (26.2)	131.3 (20.3)	0.90
HDL (mg/dl)	41.7 (6.1)	39.2 (3.4)	0.30
Triglyceride (mg/dl)	131.8 (28.2)	155.6 (25.4)	0.22
Lipoprotein (a) (mg/dl)	21.9 (17.1–39.1)	19.3 (17.9–20.6)	0.70
hsCRP (mg/l)	0.05 (0.01–0.18)	0.07 (0.01–0.16)	0.98
Fibrinogen (g/l)	3.1 (2.9–3.1)	3.1 (2.8–3.2)	0.97
PAI1 (ng/ml)	8.4 (4.5)	42.9 (3.2)	0.007
Muscle strength (kg force)	2.9 (1.9)	7.0 (1)	0.03
BMC (g)	2480.6 (488.6)	3005 (103)	0.08
BMD (g/cm^2^)	1.09 (0.09)	1.09 (0.08)	0.69
TBF (g)	32 356.4 (10 432.5)	35 908 (9146)	0.69
LBM (g)	37 072 (6545.7)	49 000 (12 071.4)	0.11
QLS-H score	45 (36.0–55.25)	41 (32.0–51.0)	0.29
Ejection fraction (%)	51.5 (49.2–52.7)	51 (3)	0.93
LV mass (g)	129.4 (22.1)	134 (16)	0.47
EDT	204 (193–219.5)	193 (186–204)	0.29
E:A ratio	1.02 (0.8–1.2)	1 (1–1.1)	0.99
E:E′ ratio	0.08 (0.07–0.08)	0.08 (0.07–0.08)	0.81

a Data presented as mean±s.d.
